# Supplementary material for: Site-specific spectroscopic measurement of spin and charge in (LuFeO3)m/(LuFe2O4)1 multiferroic superlattices
Source: Nat Commun. 2020 Nov 4;11:5582. doi: 10.1038/s41467-020-19285-9 (PMC7642375; doi:10.1038/s41467-020-19285-9)
Supplement: Supplementary file 1 — Supplementary Information [file 41467_2020_19285_MOESM1_ESM.pdf]

Supplementary Information for "Site-specific spectroscopic measurement of spin and charge in $(LuFeO_3)_m/(LuFe_2O_4)_1$ multiferroic superlattices"

Shiyu Fan, et al.

Crystal structures of the $LuFeO_3$ and $LuFe_2O_4$ end members

Supplementary Figure 1a displays the crystal structure of h-$LuFeO_3$ viewed along the [100] direction. Fe is in a trigonal bipyramidal environment surrounded by five oxygen atoms, forming Fe^{3+} polyhedra that are corner shared by the oxygen atoms in the ab-plane. The space group is $P6_3cm$, which is polar. This space group results from distortion of the non-polar $P6_3/mmc$ space group with the rotations of the FeO_5 polyhedra and Lu displacement along the c-direction[S1]. This breaks inversion symmetry and allows for polarization to develop along the c-axis. $LuFeO_3$ is thus an improper ferroelectric[S1,S2].

The other end member is $LuFe_2O_4$. The system contains Fe bilayers with both Fe^{2+} and Fe^{3+} sites. The Fe atoms are in trigonal bipyramidal environments. Prior studies reveal an antiferroelectric ground state[S3,S4] due to charge-ordering in the Fe bilayer. Supplementary Figure 1b displays the structure of the antiferroelectric state viewed along the [120] direction. Charge-ordering in the A and B bilayers are mirror images, which induces opposite electric polarizations along the c-axis. This leads to a centrosymmetric $C2/m$ space group with no net electric polarization. Lu-layer distortion is forbidden in this state.

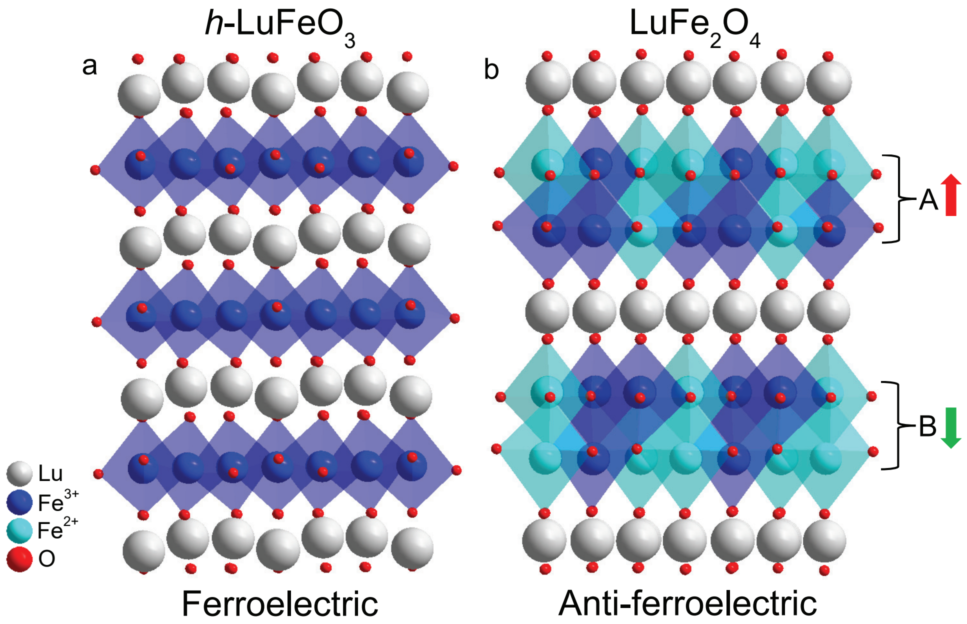

Supplementary Figure 1. **Crystal structures of the end members.** **a** Crystal structure of h-$LuFeO_3$ with the $P6_3cm$ space group. **b** Crystal structure of $LuFe_2O_4$. The light and dark blue polyhedra represent the Fe^{2+} and Fe^{3+} site, respectively.

The Fe valence in the $(LuFeO_3)_3/(LuFe_2O_4)_1$ superlattices

In prior work, we studied the Fe valence in the $LuFeO_3$ and $LuFe_2O_4$ parent compounds. We grew single phase $LuFeO_3$ films by molecular-beam epitaxy and investigated the effect of stoichiometry on the magnetic properties[S5]. Using our phase-pure samples, we demonstrated Fe^{3+} in $LuFeO_3$ samples by EELS. Annealing the samples in ozone post-synthesis did not change the observed valence or other properties, further suggesting that our synthesized samples were fully oxidized. We also note that excess Fe in these films tends to accumulate as Fe_3O_4 precipitates ($Fe^{2.67+}$) which are readily observed by magnetometry, AFM, and TEM imaging. The superlattice films used for magnetic circular dichroism spectroscopy, presented in the current manuscript, are free of these Fe_3O_4 inclusions as well.

We also explored the Fe valence in the $LuFe_2O_4$ end member by EELS[S6]. While we observed an average $Fe^{+2.5}$ valence, we were unable to identify discrete Fe^{3+} and Fe^{2+} states from a charge ordering pattern. This could be due to instrumental limitations, for instance, performing a room temperature measurement during which the electron beam averages through a column of Fe atoms. This measurement was, however, in contrast to prior work[S7] which found discrete charge-ordering. Nevertheless, our samples are consistent with a bulk valence of $Fe^{2.5+}$.

The films measured and discussed in the current manuscript were synthesized by the same MBE techniques as in our prior work. Using EELS, we again confirmed the Fe^{3+} valence in $LuFeO_3$ in the superlattices. The iron valence in the $LuFe_2O_4$ layer is slightly different than the expected 2.5+ because of the spontaneous charge transfer from the Fe^{2+} site in the $LuFe_2O_4$ layer to the Fe^{3+} site in the $LuFeO_3$ layer [Fig. 4b, main text].[S8]

At the same time, our DFT models suggest that there should be a small amount of Fe^{2+} present in the $LuFeO_3$ layers in the superlattices as a result of the ferroelectric domain walls [Fig. 4b]. Thus far, we have not been able to detect this accumulation of charge to screen the domain wall, in contrast to our ability to so in a related hexagonal magnetite system[S9]. The charge transferred to the domain wall should be approximately 0.1 e-, yielding a net valence of 2.9+ if it were to be accumulated on a single Fe site.

We can place bounds on the likely fraction of Fe^{2+} in the $LuFeO_3$ layers by examining the candidate domain wall structure in Fig. 4b. Assuming that 0.1 e^- is transferred to only the middle Fe layer in $LuFeO_3$ - as our theoretical model suggests - we find a maximum

Fe^{2+} fraction of 3%. Importantly, the overall dichroic signal from antiferromagnetic $LuFeO_3$ is much weaker than that of $LuFe_2O_4$ [Supplementary Figure 2b,c], so an additional signal from these Fe^{2+} centers in the $LuFeO_3$ layer would leave our conclusions unchanged. Our findings are therefore quite robust.

Oxygen defects can also be present in films of this type, particularly in the $LuFe_2O_4$ layer that hosts the magnetism. There is not a good lattice match for $LuFeO_3$, so while the samples are phase pure and oriented, there are threading dislocations and similar types of structural defects. Similar dislocations are also present in the $LuFeO_3$ and $LuFe_2O_4$ parent compounds in approximately the same concentration. In our prior work, we report that the magnetic moment from these defects is at least an order of magnitude smaller than that of the host compound[S5,S10]. We therefore conclude that the magnetic circular dichroic signal from these defects is extremely weak as well.

Assignments of the different electronic excitations in $(LuFeO_3)_3/(LuFe_2O_4)_1$

In the main text, we consider contributions from the three most important Fe-related excitations for the interpretation of the linear absorption spectrum and the magnetic circular dichroism. This is due to the relative size of the matrix element for the different excitations. The information on how we identify the most prominent excitations is extremely important. We list them one at a time here.

- The $Fe^{2+} \rightarrow Fe^{3+}$ charge transfer excitations are quite strong because they involve Fe sites with two different charges. Because our samples are magnetic, charge transfer in the spin-up and spin-down channels are different. These excitations appear prominently in the linear absorption as well as the magnetic circular dichroism.

- The on-site Fe^{2+} d-to-d excitation is important for the dichroic response due to the large Fe^{2+} density of states in the spin-down channel [Fig. 1c]. This excitation is also evident in the optical absorption of the $LuFe_2O_4$ end member[S10,S11].

- The Fe^{3+} $d \rightarrow d$ excitation can also take place in the $LuFe_2O_4$ layer [Fig. 1c]. This excitation is weak in the dichroic response due to antiferromagnetic behavior at the site. Moreover, the spin-up and spin-down aspects of the excitation cancel even this weak signature in the magnetic circular dichroism spectra [Fig. 1c]. This excitation

is weak in the linear absorption spectra due to the LaPorte and spin-selection rules, although symmetry-breaking activates it slightly.

- The Fe^{3+} in the $LuFeO_3$ layers is antiferromagnetic, and as a result, the intensity of the on-site Fe^{3+} d-to-d excitation is fairly modest in the dichroic response. This excitation is also weak in the linear absorption spectra due to the LaPorte and spin-selection rules, although symmetry-breaking activates it slightly.

Magnetic circular dichroism spectra of the substrate and the two end members

To obtain the dichroic response of the superlattices, we need to eliminate the substrate contribution. Supplementary Figure 2a displays the magnetic circular dichroism spectra of $(ZrO_2)_{0.905}(Y_2O_3)_{0.095}$ at ±25 T. The small, non-zero dichroic response indicates weak ferromagnetic behaviour, which is due to the small magnetic moment emanating from the Y^{3+} site. The origin of this magnetism arises as oxygen vacancies change the valency of the Y^{3+}[S12]. The sharp features in the substrate spectra at 1.57, 1.7 and 2.1 eV are likely signatures of the Y^{3+} d-to-d on-site excitations. This assignment is made based on the extremely narrow linewidth of those peaks. The temperature dependence of the substrate is negligible.

In order to extract the spectroscopic signature of the interface, we also performed magnetic circular dichroism measurements on the end members. Supplementary Figure 2b,c display the dichroic spectra of $LuFeO_3$ and $LuFe_2O_4$, respectively. The dichroic signal of the $LuFeO_3$ film is small, consistent with its antiferromagnetic nature. Nevertheless, the signal is not zero due to weak ferromagnetism induced by the slight canting of spins below 147 K[S2,S5,S13,S14]. The features centered at 1.2, 1.8, 2.1 and 2.7 eV are assigned as spin-down channel Fe^{3+} $d \rightarrow d$ on-site excitations because of the splitting in the Fe^{3+} density of states in the spin-down channel [Fig. 1c]. This assignment is also consistent with the previous literature[S15]. In contrast, ferrimagnetic $LuFe_2O_4$ has much larger dichroic signal [Supplementary Figure 2c]. Supplementary Figure 2d-f displays constant energy cut plots of the substrate, $LuFeO_3$ and $LuFe_2O_4$ at 1.33 eV, respectively. For both the substrate and $LuFeO_3$ film, $\Delta\alpha_{MCD}$ vs. magnetic field curves exhibit a quasi-linear response, indicating a non-ferrimagnetic behaviour. Note that $LuFe_2O_4$ sports a clear optical hysteresis loop, similar to the (3, 1) superlattice [Fig. 2c]. These results show that bulk magnetism in the superlattices emanates

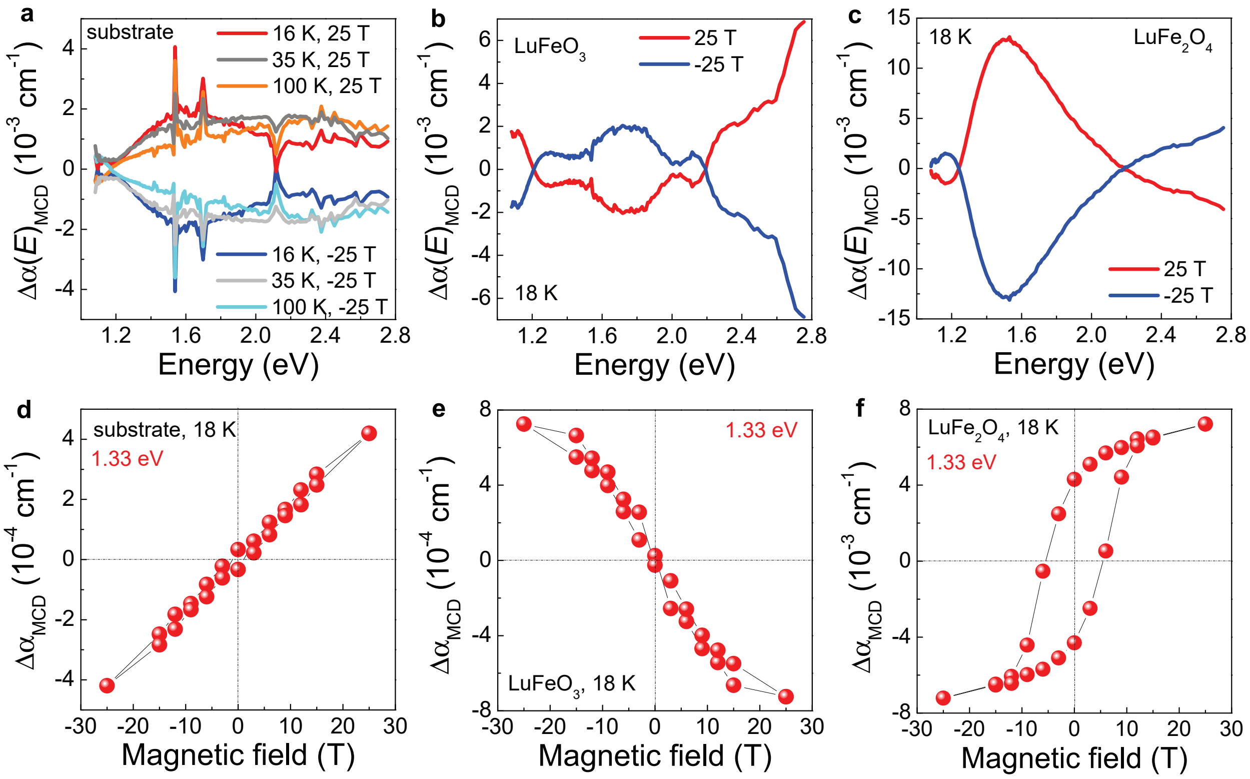

Supplementary Figure 2. **Magnetic circular dichroism spectra of the substrate and two end members, along with the corresponding optical hysteresis at 1.33 eV. a** Magnetic circular dichroism spectra of the $(ZrO_2)_{0.905}(Y_2O_3)_{0.095}$ substrate at ± 25 T at different temperatures. **b** Magnetic circular dichroism spectra of the $LuFeO_3$ at ± 25 T at 18 K. **c** Magnetic circular dichroism spectra of the $LuFe_2O_4$ at ± 25 T at 18 K. **d - f** Optical hysteresis of the substrate, $LuFeO_3$ and $LuFe_2O_4$ at 1.33 eV at 18 K, respectively.

from the $LuFe_2O_4$ layer. The coercive field of the $LuFe_2O_4$ end member is, however, much smaller than that of the superlattices. This shows that increasing Lu-layer distortion raises the single ion anisotropy of both Fe^{2+} and Fe^{3+} sites in the double layer.

Interface behavior of the superlattices

Supplementary Figure 3a displays the dichroic spectra of our set of superlattices. The $\Delta\alpha(E)_{MCD}$ exhibits a monotonic trend below 2 eV, where the dichroic intensity of the (3, 1) film is the highest. Above 2 eV, the intensity of (3, 1) superlattice drops dramatically and becomes lower than those of the (7, 1) and (9, 1) films. This behaviour is due to the inconsistent number of repeating Fe layers in the superlattices. In addition, considering

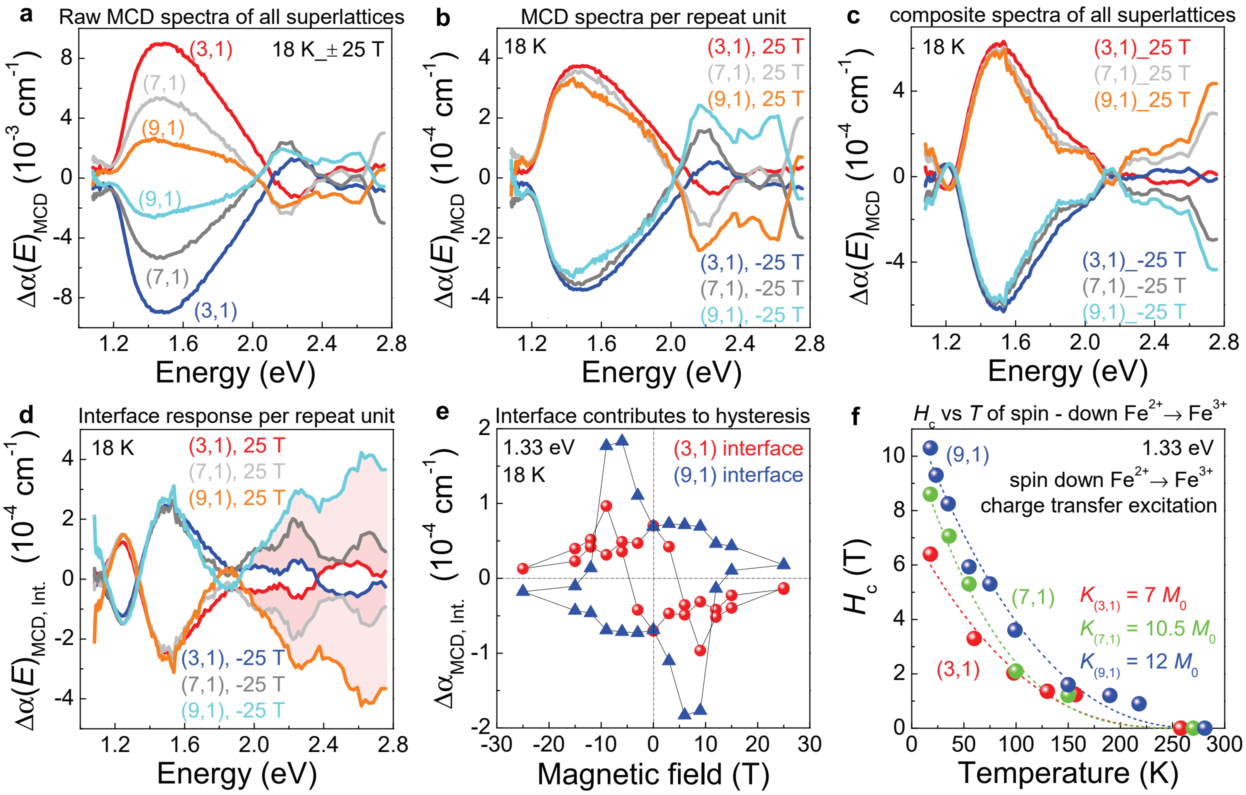

Supplementary Figure 3. **Magnetic circular dichroism spectra of the $(\mathbf{LuFeO_3})_m/(\mathbf{LuFe_2O_4})_1$ (m = 3, 7, 9), along with the composite spectra and the coercivity trends.** **a** Raw magnetic circular dichroism spectra of the (3, 1), (7, 1) and (9, 1) superlattices at ± 25 T at 18 K, before the normalization to the number of repeat units. **b** Magnetic circular dichroism spectra after normalizing to the "per repeat unit basis." **c** Composite magnetic circular dichroism spectra of all superlattices. **d** The extracted interface spectra of all the superlattics. The pink area highlights differences. **e** $\Delta\alpha_{MCD,Int.}$ of (3, 1) and (9, 1) superlattices at 1.33 eV. **f** Coercive field obtained from analysis of the spin-down channel $Fe^{2+} \rightarrow Fe^{3+}$ charge transfer excitation in the (3, 1), (7, 1) and (9, 1) superlattices vs. temperature.

the number of repeat units of the (3, 1), (7, 1) and (9, 1) superlattices are 24, 15 and 8, respectively, they contain different number of interfaces. To eliminate the contribution of the different layer thicknesses, we normalize the spectra to the "per repeat unit basis" (see Method section for details). By doing so, the magnetic behaviour of all superlattices is made comparable.

Supplementary Figure 3b displays the dichroic response per repeat unit of the (3, 1), (7,

1) and (9, 1) materials. First-principles calculations cannot be performed on higher-order films at this time due to the super-cell size, but the overall spectral shape suggests that the assignments are similar [Table 1]. We therefore employ structure-property trends to unravel the consequences of Lu-layer rumpling on the electronic structure of the interface and how these changes support nearly room temperature magnetism. Real space images reveal that increasing m strengthens the Lu-layer distortion[S16]. First-principles calculations predict that this trend amplifies the $LuFe_2O_4$ layer magnetization and dramatically boosts the ferrimagnetic T_C[S16]. We verify this overall picture by isolating the interfacial spectral response and, by so doing, unveil the underlying mechanism.

The key step in extracting the interface response is to construct the "composite spectra". Here, the composite response is simply the dichroic signal generated from combining the end member spectra based upon the composition as given by m and n. In other words, it is a simple average. The details are described in the Methods section. Supplementary Figure 3c displays the composite spectra of the (3, 1), (7, 1) and (9, 1) superlattices. There are many similarities between the composite spectra and the measured spectra [Supplementary Figure 3b], although since the composite spectra are a linear combination of the two end members, they cannot reveal the interactions between the $LuFe_2O_4$ and $LuFeO_3$ layers. In this case, by taking the difference between the experimental and composite spectra, the spectroscpic signature of the interface can be unveiled.

Supplementary Figure 3d displays the interface spectra of all the superlattices. We immediately see that $\Delta\alpha_{MCD,\,Int.}$ is significant compared to the measured dichroic response [Supplementary Figure 3b] - at least at certain energies - indicating a strong magnetic interaction between the $LuFe_2O_4$ and $LuFeO_3$ layers. Of course, the overall goal is to establish a direct link between each Fe centre and the Lu-layer distortion. We therefore take constant energy cuts of $\Delta\alpha_{MCD,\,Int.}$ and plot these values as a function of magnetic field. Supplementary Figure 3e displays the results for the (3, 1) and (9, 1) film at 1.33 eV. The wide hysteresis loop suggests that Lu-layer distortion enhances the single ion anisotropy (K) of the Fe^{2+} and Fe^{3+} sites in the spin-down channel of the Fe bilayer. Supplementary Figure 3f summarizes the extracted coercivity vs. temperature trends. We use the same model to fit the data [Fig. 2f]. The single ion anisotropy K increases with m. Magnetic anisotropy is closely related to spin-orbit coupling and crystal field strength[S17,S18]. It is therefore likely

(and consistent with Goodenough-Kamamori rules[S19]) that Lu-layer distortion modifies the Fe-O-Fe bond angle, the exchange between magnetic centres[S16], and K of Fe^{2+} and Fe^{3+} in the bilayer. Similar coercivity vs. temperature trends emerge from an analysis of the spin-up channel $Fe^{2+} \rightarrow Fe^{3+}$ charge-transfer [Fig. 3b] and the spin-down channel Fe^{2+} $d \rightarrow d$ excitations, revealing an overall stronger single ion anisotropy in the $LuFe_2O_4$ layer with increasing Lu-layer distortion.

Optical hysteresis at characteristic energies

One important challenge in this work is to separate the magnetic contribution of a specific Fe site from multiple Fe centres. The magnetic signature of $Fe^{2+} \rightarrow Fe^{3+}$ charge-transfer excitation in the spin-down channel is explicitly observed by cutting the dichroic spectra at 1.33 eV. Nonetheless, the signatures of $Fe^{2+} \rightarrow Fe^{3+}$ charge-transfer excitation in the spin-up channel and Fe^{2+} $d \rightarrow d$ on-site excitations in the spin-down channel are difficult to extract because of mixing above 2 eV. Here, we explain the process of separating the responses of different Fe-related excitations and report the peculiar appearance of the raw data when the different contributions are superimposed.

Supplementary Figure 4a-c displays three different energy cuts at 1.8, 2.2 and 2.6 eV from the raw magnetic circular dichroism spectra. At 1.8 eV, the optical hysteresis displays a similar shape to the 1.33 eV loop (Fig. 2c), revealing that the excitation at 1.8 eV is relatively unmixed. In contrast, at 2.2 eV, the shape of the hysteresis loop becomes highly irregular, because of the superposition of two individual optical hysteresis loops. Since the dichroic intensities of the interface at 1.8 and 2.2 eV are relatively similar [Fig. 3a], we subtract the 1.8 eV loop from the loop at 2.2 eV to extract the pure magnetic signature of $Fe^{2+} \rightarrow Fe^{3+}$ charge-transfer excitation in the spin-up channel. Similar analysis is extended to the optical hysteresis loop at 2.6 eV, where the shape exhibits the irregularities again because of the mixing between charge-transfer and d-to-d excitations. We subtract the response of the $Fe^{2+} \rightarrow Fe^{3+}$ charge-transfer excitation in the spin-up channel from the hysteresis loop at 2.6 eV. The result displays the pure response of Fe^{2+} $d \rightarrow d$ excitation [Fig. 2e].

We performed a similar analysis for the (7, 1) and (9, 1) superlattices. Supplementary Figure 4d-f displays the optical hysteresis on a "per repeat unit" basis for the $Fe^{2+} \rightarrow Fe^{3+}$ charge-transfer excitations in both spin-down and spin-up channels and the Fe^{2+} $d \rightarrow d$

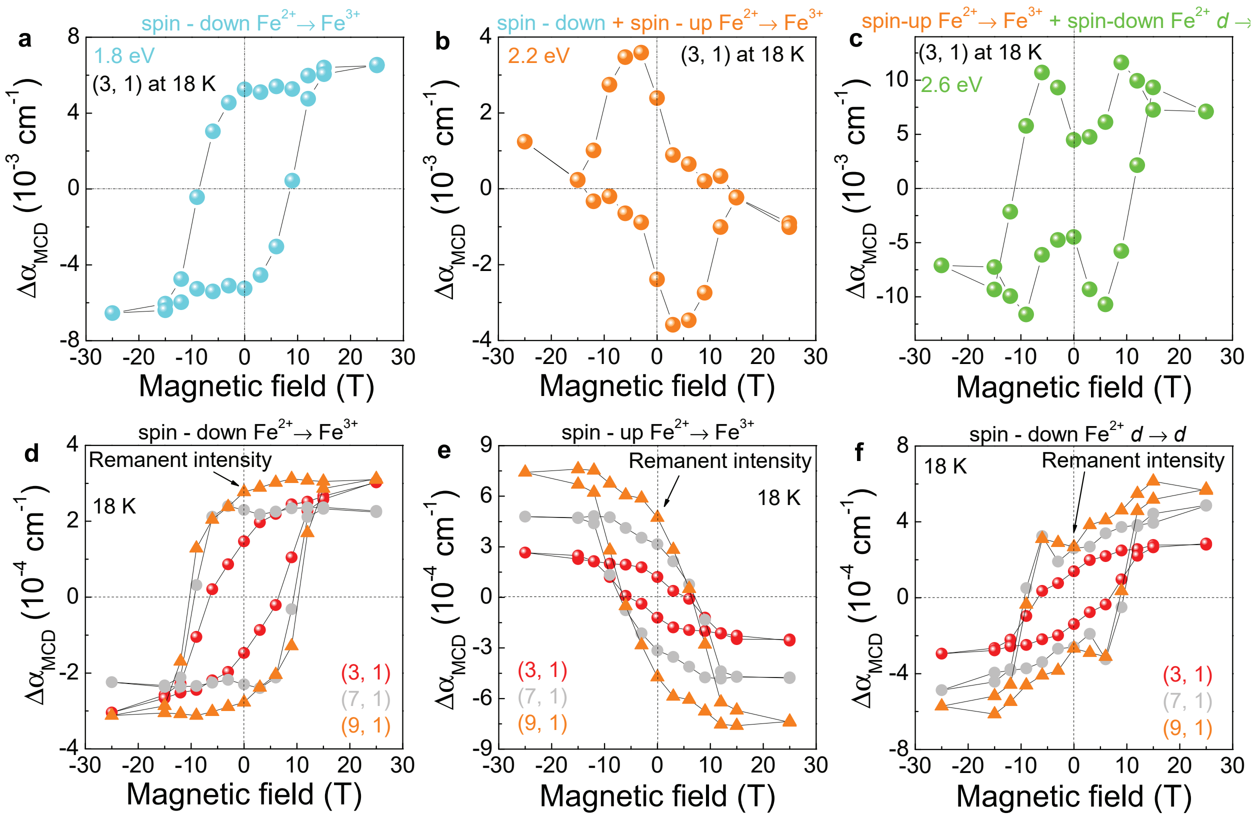

Supplementary Figure 4. **Optical hysteresis at characterisic energies. a - c** Optical hysteresis at 1.8, 2.2 and 2.6 eV of the (3, 1) superlattice. The data points are directly obtained from the constant energy cuts of the raw spectra. **d** Optical hysteresis loops of (3, 1), (7, 1) and (9, 1) superlattices at 1.33 eV at 18 K. **e** Optical hysteresis of the spin-up channel $Fe^{2+} \to Fe^{3+}$ charge-transfer excitation at 18 K. **f** Optical hysteresis of the spin-down channel Fe^{2+} $d \to d$ on-site excitation at 18 K.

on-site excitation in the spin-down channel. At the same energy, the optical hysteresis of all superlattices exhibit similar ferrimagnetic behaviour, except for width and height differences. The width indicates the coercive field, which increases with the number of $LuFeO_3$ layers. As discussed before, this behaviour reveals increasing magnetic anisotropy with increasing Lu-layer distortions. The height of each hysteresis loop illustrates the remnant dichroic intensity ($\Delta\alpha_{MCD,\,Rem}$), which is directly related to the remnant magnetization[S21,S22]. The results clearly reveal that remnant magnetization rises with increasing the Lu-layer distortion for all types of Fe-related excitations. In addition, the magnitude of the remnant magnetization of the $Fe^{2+} \to Fe^{3+}$ charge-transfer excitation in the spin-up channel [Supplementary Figure

4e] increases the most. The quantitative $\Delta\alpha_{MCD,\ Rem}$ vs Lu-layer distortion trend is displayed in Fig. 3c. Notice that there are still some irregularities related to the loops associated with Fe^{2+} $d \rightarrow d$ excitations of higher order films ($m = 7$ and 9), probably due to enhanced interface effects at higher energies that rise with Lu-layer distortion [Fig. 3a].

Developing interface design rules for increasing T_C and enhancing magnetoelectric coupling

In order to develop a heuristic argument for why the spin-up channel charge transfer excitation is so important in the $(LuFeO_3)_m/(LuFe_2O_4)_n$ superlattices, we sought to identify which orbitals are active in the Fe double-layer. To do so, we consider a slab of $LuFe_2O_4$ surrounded by $LuFeO_3$. Each Fe center - regardless of charge - is in a trigonal bipyramidal geometry. Given this local structure, the $d_{x^2-y^2}$ and d_{xy} orbitals are in-plane [Supplementary Figure 5a]. They are also degenerate and orthogonal. We hypothesize that the in-plane orbital arrangement in the spin-up channel is responsible for the increasingly robust magnetism [Fig. 3b] and higher T_C in the (3, 1) → (7, 1) → (9, 1) series. How the overlap and hybridization change in response to distortion of the Lu layer is likely to impact magnetoelectric coupling as well.

Supplementary Figure 5b,c displays the in-plane orbitals in a hypothetical $LuFe_2O_4$ slab. The top-down view emphasizes the honeycomb arrangement of the Fe^{2+} and Fe^{3+} centres, the $d_{x^2-y^2}$ and d_{xy} orbitals, and their overlap with the O p_x and p_y orbitals. The side-on schematic - with its simplified linear arrangement of polyhedra and lack of O p_x and p_y orbitals - allows us to clearly see the structural distortions that take place in the bilayer. Examination reveals that Lu rumpling has an important effect on the overlap of the in-plane orbitals. This is due to the tendency of the Fe^{3+}-containing trigonal bipyramids to rotate and elongate in response to the Lu-layer distortion. The Goodenough-Kanamori rules govern how local structure distortions modify orbital overlap and exchange interactions[S19]. Based upon the modification of orbital overlap and hybridization, these rules qualitatively explain trends in the (3, 1), (7, 1), and (9, 1) series.

We already know from STEM and first principles calculations that larger m yields greater Lu-layer distortion and higher T_C [Fig. 1][S16]. Our analysis suggests that the Lu-layer distor-

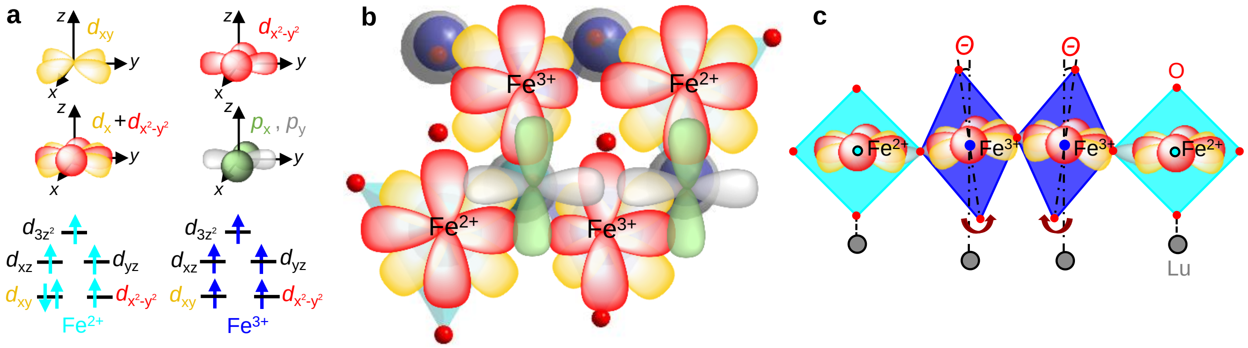

Supplementary Figure 5. **Schematic view of the in-plane orbital overlap and the consequences of Lu-layer distortion.** **a** Schematic view of the Fe d_{xy} and $d_{x^2-y^2}$ orbitals + the O p_x and p_y orbitals. The ground state energy diagrams for the Fe^{2+} and Fe^{3+} sites are shown as well. **b** Top view of the $LuFe_2O_4$ double layer in the self-doped structure showing how tilting of the FeO_5 trigonal bipyramids modifies hybridization. This changes the overlap of the in-plane orbitals. **c** Schematic side-on view of a hypothetical linear array of FeO_5 trigonal bipyramids and the rotation and elongation that result from Lu-layer distortion. The tilting and elongation is highly exaggerated in this rendering, and the O p_x and p_y orbitals are omitted for simplicity.

tion causes the Fe^{3+} polyhedra in the $LuFe_2O_4$ double layer to elongate and rotate, changing the in-plane orbital overlap in such a way as to make the spin-up charge transfer more important. Therefore, by controlling the atomistic details of the rotation, one can influence hybridization. The structurally-induced orbital reconstruction modifies the net magnetic moment on the Fe^{3+} sites such that bilayer magnetism becomes more robust. This raises the coercive field [Fig. 3b] and the magnetic Curie temperature.

One consequence of an elongated polyhedron is a possible off-mirror plane distortion of the Fe^{3+} centers. Such a distortion was recently observed in the ferrimagnetic quantum paraelectric $BaFe_{12}O_{19}$[S20]. Our STEM images provide an upper bound of $\leq$ 10 - 20 pm to any Fe^{3+} off-centering in the $(LuFeO_3)_m/(LuFe_2O_4)_1$ superlattices. This level of off-centering is negligible and does not contribute to the properties of these materials.

Determining the charge-ordered state of bulk $LuFe_2O_4$

The end member $LuFe_2O_4$ is well known for its complex charge-ordering states because it determines the ferroelectricity in the material [Supplementary Figure 6a, b]. Previous

calculations predict two nearly degenerate charge-ordered states (CO-I and CO-II) in bulk $LuFe_2O_4$[S3,S7,S16,S23,S24], where the energy of the CO-II structure is only 4 meV per formula unit higher than the CO-I structure[S16]. CO-I is an antiferroelectric state in which the Lu-layer trimer distortion is forbidden by symmetry. In the CO-II state, by contrast, the first Fe layer (A) is composed of a 1:2 ratio of Fe^{2+} and Fe^{3+} centres, whereas the second Fe layer (B) has a similar charge configuration but with a majority of Fe^{2+}. This $ABAB$-type stacking breaks inversion symmetry and brings ferroelectricity to the system. Lu-layer trimer distortion is allowed in the CO-II state[S16].

In order to reveal the relative importance of the charge-ordered states and potentially discriminate between them, we calculated magnetic circular dichroism using first-principles methods and compared the results to the experimental dichroic spectra. To accomplish this,

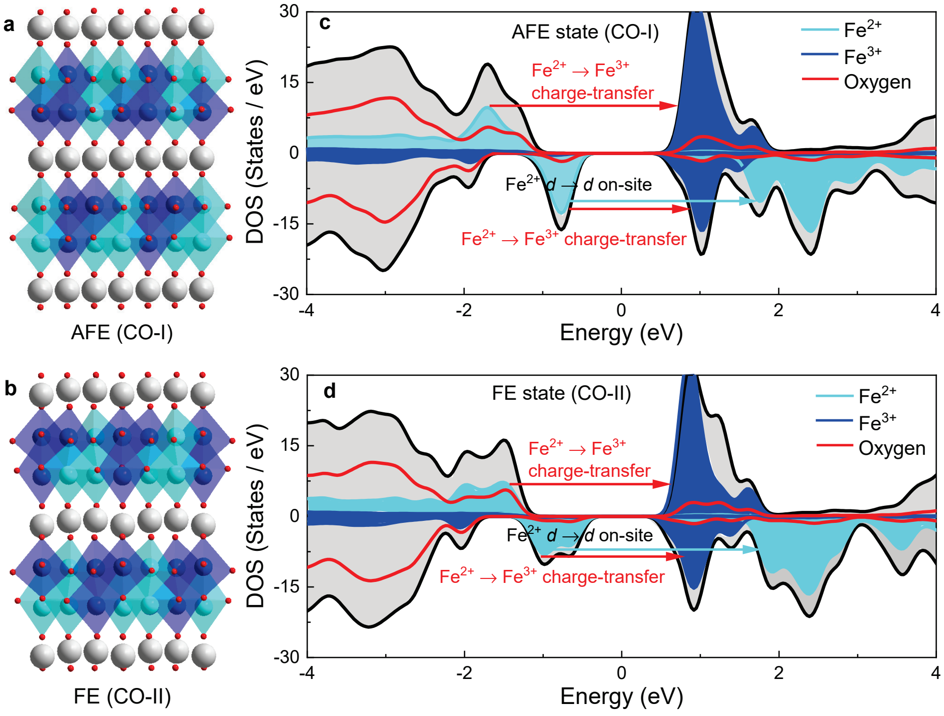

Supplementary Figure 6. **Computed density of states of $LuFe_2O_4$ system. a, b** Optimized crystal structures of the CO-I and CO-II charge-ordering states, respectively. The light and dark blue polyhedra indicate the crystal environment of Fe^{2+} and Fe^{3+} centers, respectively. **c, d** Calculated partial density of states corresponding to the CO-I and CO-II charge-ordering patterns, respectively.

we first compute the density of states of the CO-I and CO-II structures [Supplementary Figure 6c, d]. The Fe density of states are almost the same, but the energy of the Fe-related excitations are slightly shifted. This causes the different peak positions in the dichroic response. The excitation assignments here are slightly different compared to those in the (3, 1) superlattice, because the Lu-layer distortion at the interface shifts the energies of both Fe^{2+} and Fe^{3+} density of states in the $LuFe_2O_4$ layer.

Supplementary Figure 7a,b displays the calculated and measured dichroic spectra of the end members. Overall, the computed spectra are in reasonable agreement with the measured response. Specifically, the calculated spectrum of $LuFeO_3$ shows excellent agreement with the experimental results, except for the missing shoulder at approximately 1.2 eV. For $LuFe_2O_4$, $\Delta\alpha(E)_{MCD}$ of the CO-I state is in much better agreement with the experimental data. The distinction is particularly strong above 1.5 eV where the spectra corresponding to the CO-I and CO-II states diverge. Interestingly, this is the energy window of the $Fe^{+2} \rightarrow Fe^{+3}$ charge-transfer excitation in the spin-down channel. We also find that the Fe^{+2} $3d$ states are more localized in the CO-I state compared with the ferroelectric CO-II state. Our calculations predict a dip in the spectra near 2.0 eV where the Fe^{+2} and Fe^{+3} $d \rightarrow d$

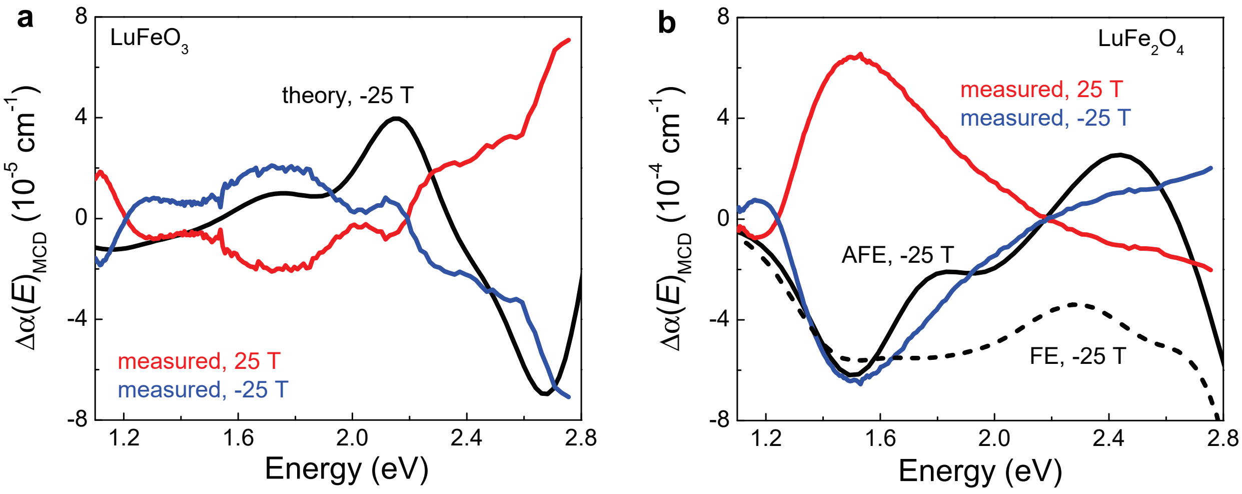

Supplementary Figure 7. **The calculated vs. measured dichroic spectra of the end members. a, b** Computed and measured magnetic circular dichroism (MCD) spectra for the $LuFeO_3$ and $LuFe_2O_4$ end members, respectively. The calculated MCD spectra for the CO-I (AFE) and CO-II (FE) states are indicated by solid and dashed lines, respectively. AFE and FE refer to antiferroelectric and ferroelectric, respectively.

excitations begin - although this feature is not replicated in the experimental data. In any case, our analysis indicates that $LuFe_2O_4$ is best represented by the antiferroelectric CO-I model [Supplementary Figure 7b] - a finding that is in agreement with the literature[S3,S4,S16]. The presence of a net electric polarization in the CO-II state may have a role to play behind the observed differences between the two models - a subject of future work.

[S1] W. Wang., et al. Room-temperature multiferroic hexagonal $LuFeO_3$ films. Phys. Rev. Lett. **110**, 237601 (2013).

[S2] Disseler, S. N., et al. Magnetic structure and ordering of multiferroic hexagonal $LuFeO_3$. Phys. Rev. Lett. **114**, 217602 (2015).

[S3] Angst, M. et al. Charge order in $LuFe_2O_4$: antiferroelectric ground state and coupling to magnetism. Phys. Rev. Lett. **101**, 227601 (2008).

[S4] Xu, X. S., et al. Lattice dynamical probe of charge order and antipolar bilayer stacking in $LuFe_2O_4$. Phys. Rev. B. **82**, 014304 (2010).

[S5] Moyer, J. A. et al. Intrinsic magnetic properties of hexagonal $LuFeO_3$ and the effects of nonstoichiometry. APL Mater. **2**, 012106 (2014).

[S6] Mundy, J. A. et. al. Atomic-resolution chemical imaging of oxygen local bonding environments by electron energy loss spectroscopy. Appl. Phys. Lett. **101**, 042907 (2012).

[S7] Ikeda, N., et al. Ferroelectricity from iron valence ordering in the charge-frustrated system $LuFe_2O_4$. Nature. **436**, 1136 (2005).

[S8] Holtz, M. E. et al., Dimensionality-induced change in topological order in multiferroic oxide superlattices. Preprint at arXiv:2007.03872 [cond-mat.mtrl-sci] (2020).

[S9] Mundy, J. A. et al. Functional electronic inversion layers at ferroelectric domain walls. Nature Mater. **16**, 622 (2017).

[S10] Brooks, C. M. et al. The adsorption-controlled growth of $LuFe_2O_4$ by molecular-beam epitaxy. Appl. Phys. Lett. **101**, 132907 (2012).

[S11] Xu, X. S. et al. Charge order, dynamics, and magnetostructural transition in multiferroic $LuFe_2O_4$. Phys. Rev. Lett. **101**, 227602 (2008).

[S12] Bayati, R., et al. Modification of properties of yttria stabilized zirconia epitaxial thin films

by excimer laser annealing. Appl. Mater. Interfaces. **6**, 22316 (2014).

[S13] Das, H., Wysocki, A. L., Geng, Y., Wu, W., and Fennie, C. J. Bulk magnetoelectricity in the hexagonal manganites and ferrites. Nat. Commun. **5**, 2998 (2014).

[S14] Cao, S., et al. The stability and surface termination of hexagonal $LuFeO_3$. J. Phys.: Condens. Matter. **27**, 175004 (2015).

[S15] Holinsworth, B. S., et al. Direct band gaps in multiferroic h-$LuFeO_3$. Appl. Phys. Lett. **106**, 082902 (2015).

[S16] Mundy, J. A. et al. Atomically engineered ferroic layers yield a roomtemperature magnetoelectric multiferroic. Nature. **537**, 523 (2016).

[S17] Rinehart, J. D. and Long J. R. Exploiting single-ion anisotropy in the design of f-element single-molecule magnets. Chem. Sci. **2**, 2078 (2011).

[S18] Atanasov, M., Zadrozny, J. M., Long, J. R., and Neese, F. A theoretical analysis of chemical bonding, vibronic coupling, and magnetic anisotropy in linear iron(II) complexes with single-molecule magnet behavior. Chem. Sci. **4**, 139 (2013).

[S19] Goodenough, J. B. Magnetism and the chemical bond. Interscience-Wiley, New York. (1963).

[S20] Cao, H. B., et al. High pressure floating zone growth and structural properties of ferrimagnetic quantum paraelectric $BaFe_{12}O_{19}$. APL Mater. **3**, 062512 (2015).

[S21] Dobrowolska, M., et al. Controlling the Curie temperature in (Ga,Mn)As through location of the Fermi level within the impurity band. Nat. Mater. **11**, 444-449 (2012).

[S22] Rice, W. D., et al. Persistent optically induced magnetism in oxygen-deficient strontium titanate. Nat. Mater. **13**, 481 (2014).

[S23] Xiang, H. J., and Whangbo, M. -H. Charge order and the origin of giant magnetocapacitance in $LuFe_2O_4$. Phys. Rev. Lett. **98**, 246403 (2007).

[S24] Nagano, A., Naka, M., Nasu, J., and Ishihara, S. Electric polarization, magnetoelectric effect, and orbital state of a layered iron oxide with frustrated geometry. Phys. Rev. Lett. **99**, 217202 (2007).
